# Preparation and Characterization of Electrosprayed Aerogel/Polytetrafluoroethylene Microporous Materials

**DOI:** 10.3390/polym14010048

**Published:** 2021-12-23

**Authors:** Xiaoman Xiong, Mohanapriya Venkataraman, Tao Yang, Jiří Militký, Jakub Wiener

**Affiliations:** 1 Department of Material Engineering, Faculty of Textile Engineering, Technical University of Liberec, 46117 Liberec, Czech Republic; mohanapriya.venkataraman@tul.cz (M.V.); jiri.militky@tul.cz (J.M.); jakub.wiener@tul.cz (J.W.); 2 Institute for Nanomaterials, Advanced Technologies and Innovation, Technical University of Liberec, 46117 Liberec, Czech Republic; tao.yang@tul.cz

**Keywords:** electrospray, polytetrafluoroethylene, aerogel, surface roughness, hydrophobicity, thermal conductivity

## Abstract

This paper presents the preparation of aerogel/polytetrafluoroethylene (PTFE) microporous materials via needleless electrospray technique, by using an aqueous dispersion of polytetrafluoroethylene as the basic spinning liquid. Different contents of aerogel powders were applied to the spinning liquid for electrospraying to investigate the effect on the structural characteristics and various properties of the materials. Cross-section, surface morphology, and particle size distribution of the electrosprayed materials were examined. Surface roughness, hydrophobicity, and thermal conductivity were evaluated and discussed. The results showed that the electrosprayed aerogel/PTFE layers were compact and disordered stacking structures composed of spherical particles with a rough surface. As the aerogel content increased, the electrosprayed materials demonstrated increased surface roughness and improved surface hydrophobicity with a contact angle up to 147.88°. In addition, the successful achievement of thermal conductivity as low as 0.024 (W m^−1^ K^−1^) indicated a superior ability of the prepared aerogel/PTFE composites to prevent heat transfer. This study contributes to the field of development of aerogel/PTFE composites via electrospray technique, providing enhanced final performance for potential use as thermal and moisture barriers in textiles or electronic devices.

## 1. Introduction

As a synthetic fluoropolymer with a general formula of $\left[{\left(-CF_{2}-\right)}_{n}\right]$, polytetrafluoroethylene (PTFE) demonstrated excellent chemical resistance, low surface energy, desirable dielectrical properties, strong hydrophobicity, low flammability, and good thermal stability [1,2,3,4]. These characteristics made it widely used as a functional polymeric material in non-stick coating, lubrication, bearing balls, insulating applications in cables, electronic devices, and heat management [4,5,6]. The pure PTFE materials have a thermal conductivity of around 0.3 W m^−1^ K^−1^) [7], much higher than conventional and advanced thermal-insulating materials, but lower than the requirement of a heat exchanger in process industries, which limits its engineering applications. Currently, research on improvements in the thermal conductivity of PTFE, by adding fillers such as graphite and ceramics is very advanced [8,9], while studies focusing on the enhancement of its thermal insulation are comparatively weak. PTFE has been composited with various fillers to tune the properties; the intrinsic properties of the polymer and filler, the shape, content, size, and mass fraction of the filler, strongly influence the properties of the composites [10,11]. Silica aerogel is an ideal super-insulating material to incorporate with PTFE to improve the thermal insulating property.

Silica aerogel is a coherent, rigid three-dimensional network of contiguous particles of colloidal silica [12]. Silica aerogels possess a special microstructure with pore characteristic size ranging from 2 to 50 nm, and more than 90% of its overall volume is occupied by air [13]. Aerogels demonstrate extremely low thermal conductivity, low bulk density, high specific surface area, and some other characteristics [14,15]. However, due to the highly open structures in which the secondary particles of silica are connected to each other with only few siloxane bonds, silica aerogels generally have low strength and high brittleness. Therefore, aerogels are usually used in the form of particles as fillers in composite with other materials. Aerogel particles have been thermally bonded in polyester nonwovens [16], encapsulated in multilayered fibrous materials [17], and embedded in PUR and PVDF nanofibrous layers by electrospinning [18], achieving lower thermal conductivity of the overall structures.

There is a large volume of published studies describing the attempts to incorporate aerogels with PTFE materials. Silica aerogel reinforced PTFE composites with 10–30 wt% silica aerogel have been synthesized via mixing hydrophobic silica aerogel powders and PTFE powders in absolute alcohol, pressing the mixture to harden the composites and sintering [19]. By utilizing this method, PTFE was successfully filled into the pores of aerogel particles, along with chemical reactions between the two materials. Analysis of thermal behavior found that thermal conductivity of the composite decreases obviously with the increase in aerogel content when the value is less than 18 wt%, while above 18 wt% the decrease rate is fairly small. The same approach was utilized in Yung-Chih Chen’s study, where the phenyltrimethoxy silane-coated SiO_2_ powder and aqueous PTFE dispersion were used as starting materials instead [11]. The effects of SiO_2_ content and particle size on the thermal, dielectric, and mechanical properties of PTFE/SiO_2_ composites were discussed. Another similar work conducted by K.P. Murali et al. was focused on microwave circuit applications. In their study, silica filled PTFE materials with varying filler loading were prepared through sigma mixing, extrusion, and calendering followed by hot pressing [20]. Xiaoliang Wei reported a novel PTFE/silica nanoporous composite in which the PTFE particles were fibrillated to form a PTFE matrix [21]. This composite was created via shear-blending the mixture of aqueous PTFE dispersion and amorphous precipitated silica nano-particles at elevated temperatures, providing excellent electrochemical performance and proven chemical stability. A methylsilsesquioxane aerogel coating layer fabricated on ePTFE thin film was described by Xingzhong Guo, the flexible and lightweight material prepared via a simple blade coating process showed good dielectric property and electrical insulation property [22]. Although these studies have successfully incorporated aerogels with PTFE, the involved fabrication methods bring about high energy consuming and environmental pollution problems since the casting of PTFE materials is mostly conducted by a molding technique [23]. Moreover, it is challenging to create a desirable continuous aerogel/PTFE layer for applications when light weight and miniaturization are essential.

In this work, a more convenient and flexible method, the needleless electrospray technique, was employed to composite silica aerogels with PTFE by using an aqueous dispersion of polytetrafluoroethylene as the basic spinning liquid. Electrospray produces small monodisperse particles, in the form of a thin film of fine particles, from a colloidal suspension of solid nanoparticles or a solution of a material. In a large electric field, as the electric field becomes stronger, the highly-charged droplet held on the capillary tip or a surface [24] by surface tension deforms into a Taylor cone jet. If the electric repulsive forces are strong enough to overcome the surface tension, a continuous fluid will be emitted to generate a spray. Since the spinning fluid has sufficiently low viscosity, the jet disintegrates downstream and subsequently breaks into charged droplets [25]. These droplets are sub-micrometer or micrometer in diameter and they rapidly evaporate due to their large surface-to-volume ratio, forming electro-sprayed particles on the substrate material. Electrospray enables the use of various fillers for performance modification or functionalization, which provides more possibility for the development of PTFE-based porous materials or composites.

The prepared materials were characterized in terms of surface morphology, particle size distribution, structural characteristics, as well as properties including surface roughness, hydrophobicity, and thermal conductivity. The effect of aerogels on these properties was determined and is discussed in detail. The novelty of this study lies in using the nano-spray technique to prepare aerogel/PTFE microporous material, the electrosprayed materials achieved a significantly decreased thermal conductivity and improved surface hydrophobicity, which have the potential to be used as a thermal and moisture barrier in textiles or electronic devices for protection against heat and moisture.

## 2. Materials and Methods

### 2.1. Materials

An aqueous dispersion of fluoropolymer Teflon^TM^ PTFE DISP 30 (without kyselina perfluoroktanová), obtained from Chemours, Wilmington, NC, USA, was used for electrospray. It contains 60% by weight of polytetrafluoroethylene with typical particle size of 0.220 μm, suspended in distilled water, together with a 6% by weight surfactant. The density of the dispersion is 1.51 g cm^−3^, the viscosity at 25 °C is 25 MPa s. Silica aerogel, in the form of powders, was selected as filler to modify the properties of the electrosprayed PTFE materials. Aerogels may influence the structure and composition of the electrosprayed materials and thus affect the behavior of the materials. Aerogels (Cabot aerogel Corp., Boston, MA, USA) used in this study have a majority particle size ranging from 2 to 40 μm. The thermal conductivity of the aerogels is 0.012 W m^−1^ K^−1^) at 25 °C.

### 2.2. Preparation of Electro-Sprayed PTFE/Aerogel

**Figure 1:**
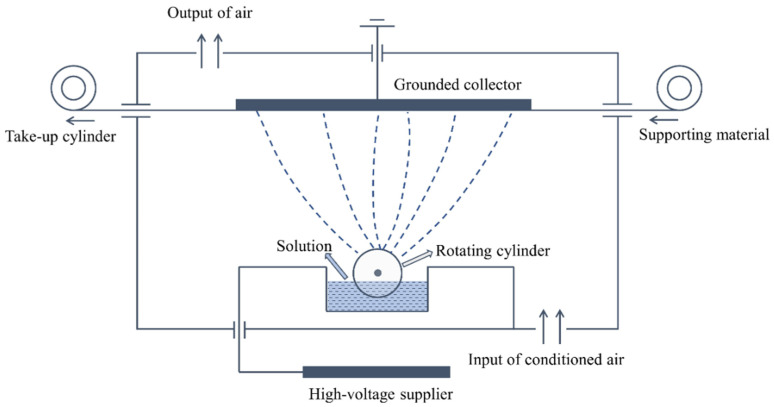
Schematic setup of electrospraying.

A certain quantity of aerogels was applied in the PTFE dispersion as received, the mixture was stirred for 2 h at room temperature (around 25 °C) to achieve a homogeneous solution and avoid any visible deposition. After that, the mixture was delivered to the Nanospider instrument NS 1WS500U (Elmarco Inc., Morrisville, NC, USA) for electrospraying as shown in Figure 1.

The electrostatic force produced between the high-voltage supplier and the grounded collector drew the liquid from the surface of the electrode in the form of a rotating cylinder, the charged jet then broke down into small droplets that solidified during the course to the collector and were deposited on the surface of the support material [26]. The rotating cylinder kept the ongoing electrospray process to continuously produce the particles, and thus an even layer of electro-sprayed particles was obtained with coordination of the movement of the support material. Descriptions of the prepared materials as well as the composition of each solution used for electrospray are listed in Table 1. Sample S0 consisted only of PTFE microparticles, while other samples contain both PTFE particles and aerogels.

To achieve the optimized conditions, solutions were prepared and trialed on the Nanospider device to set the spinning parameters before formal fabrication. The adjusting of process parameters was necessary for the needleless electrospinning system of Nanospider to prepare layers with controllable porosity. The optimized parameters are given in Table 2.

### 2.3. Characterization

#### 2.3.1. Morphologies

The scanning electron microscope VEGA TESCAN (TESCAN Inc., Warrendale, PA, USA) was employed to observe the cross-section and surface morphologies of the electrosprayed materials. SEM images were obtained after coating the samples with a gold film. The cross-section image enables viewing the multilayered structure of the materials, while the surface morphology image of each material provided detailed information about the electrosprayed particles, such as the grain structures and features.

#### 2.3.2. Particle Size Distribution

To evaluate the particle size distribution of the electrosprayed particles, ImageJ software (National Institute of Mental Health, Bethesda, MD, USA) was used to conduct image analysis on the obtained SEM images. Image analysis is a convenient and effective approach to detect grain size and size distribution with reliable accuracy, which is widely used in various scientific fields to deal with morphological studies.

#### 2.3.3. Thickness and Areal Density of the Materials

The thickness of the electrosprayed materials was measured by a fabric thickness tester DIN 878 (SOMET CZ s.r.o., Bílina, Czech Republic), following the standard ASTM D1777-15. The areal density (GSM) of each material was determined according to the ASTM D3776-96 standard.

#### 2.3.4. Surface Roughness

The surface roughness of the electrosprayed materials was evaluated by a Measuring Laser Microscope OLS5000 LEXT (Olympus America Inc., Webster, TX, USA). By scanning laser light over the sample surface, this microscope provided enlarged images of microscale features to perform shape measurements of surface roughness and other features.

Based on the scanned image, every ten different lines were recorded and then calculated automatically into the main roughness parameters including arithmetic average height *R_a_*, maximum peak height *R_p_*, maximum valley depth *R_v_*, and skewness *R_sk_*.

**Figure 2:**
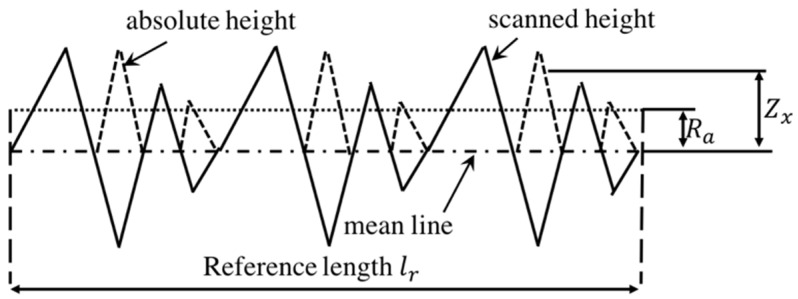
Description of measuring surface roughness [27].

As seen in Figure 2, *R_a_* [μm] is the mean absolute value of the individual height deviation from the arithmetic mean elevation of the profile within the evaluation length, given by

(1) $$R_{a}=\frac{1}{l_{r}}\int_{0}^{l_{r}}\left|Z\left(x\right)\right|dx$$

where *x* is the direction of calculation, *l_r_* is the reference length along the *x*-direction, and *Z*(*x*) is the height deviation from the mean level at *x* position. The third standardized moment *R_sk_* is a measure of the skewness, i.e., the departure of the height deviation distribution from symmetry

(2) $$R_{sk}=\frac{1}{R_{q}^{3}}\left(\frac{1}{l_{r}}\int_{0}^{l_{r}}Z^{3}\left(x\right)dx\right)$$

where *R_q_* [μm] is the standard deviation of height deviation distribution (square root of the sum of the squares of the deviation of the individual heights defined as

(3) $$R_{q}=\sqrt{\frac{1}{l_{r}}\int_{0}^{l_{r}}Z^{2}\left(x\right)dx}$$

A negative value of *R_sk_* indicates that the surface profile has more flat valleys, whereas a surface profile with a positive skewness contains mainly peaks and asperities.

#### 2.3.5. Water Contact Angle

The water contact angle is a quantitative measure of wetting of a solid by water. The contact angle is geometrically defined as the angle formed by a liquid at the three-phase boundary where a liquid, gas, and solid intersect. A computer-based instrument named “A see system E” (Advex Instruments, s. r. o., Brno, Czech Republic) was utilized to evaluate the contact angle, following the ISO 27448:2009 standard. A microsyringe (5 µL) was employed to drop deionized water onto the material, a picture of the drop was taken in 1 min to be displayed in the PC for calculating the contact angle. Five drops were measured for each sample.

#### 2.3.6. Thermal Conductivity

The Alambeta Instrument (SENSORA, Liberec, Czech Republic) was used to measure the thermal conductivity of the electrosprayed materials according to the EN 31092 Standard. The measuring head of the Alambeta contains a copper block, which is electrically heated to approximately 32 °C to simulate human skin temperature, the lower part of the heated block is equipped with a direct heat flow sensor which measures the thermal drop between the surfaces of a very thin nonmetallic plate using a multiple differential microthermocouple [28]. Based on Fourier’s law of heat conduction, thermal conductivity value was automatically calculated and shown on the display screen.

## 3. Results and Discussion

### 3.1. Cross-Section and Surface Morphology of the Electro-Sprayed Materials

**Figure 3:**
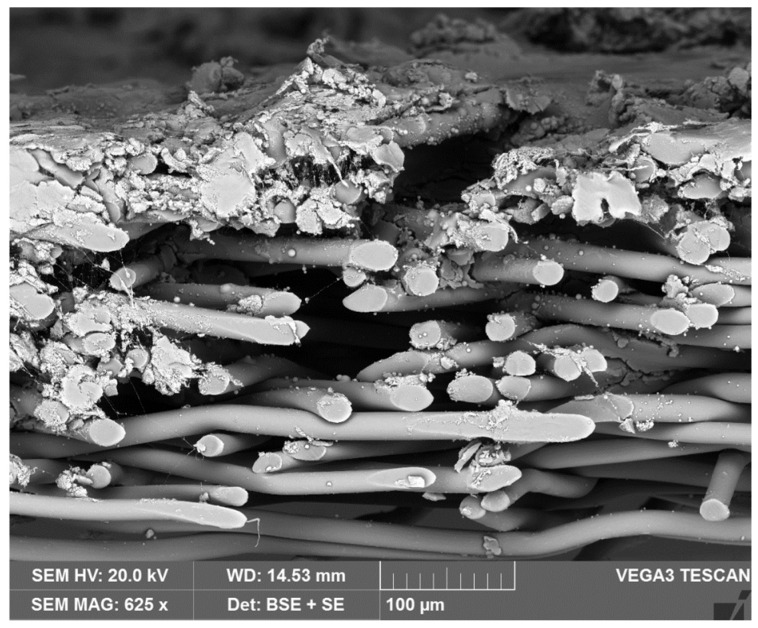
Cross-section image of the prepared material.

A typical cross-section image of the prepared material is illustrated in Figure 3. The lower part of the image shows the spun-bond nonwoven substrate and the upper part shows the structure of the deposit generated by the PTFE and aerogel particles. The electrosprayed particles were successfully deposited on the nonwoven substrate, forming a multilayered cross-section structure. The nonwoven substrate was inherently highly porous with numerous interconnected open pores on the surface, therefore, some electro-sprayed particles filled these voids between the fibers instead of simply attaching on them to the nonwoven surface.

**Figure 4:**
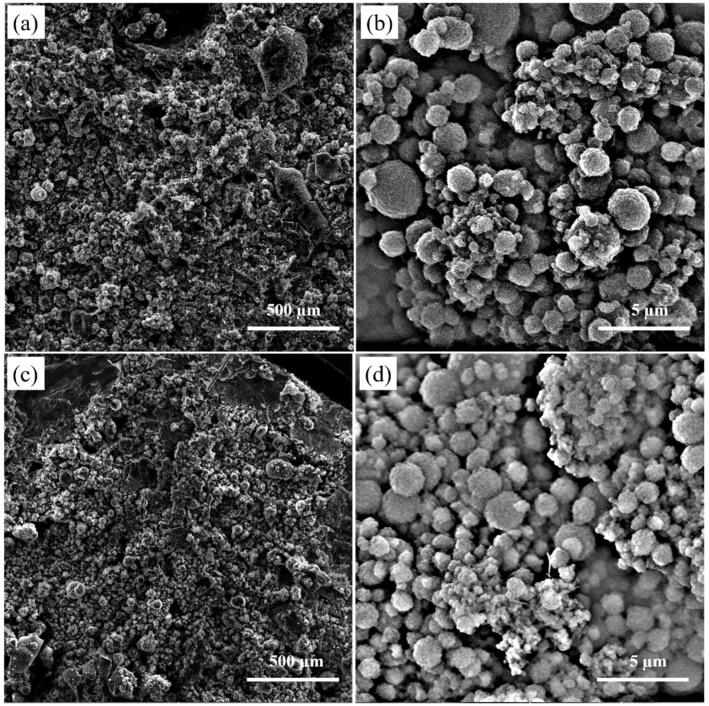
SEMs of the electrosprayed materials (**a**)—sample S0 with low magnification; (**b**)—sample S0 with high magnification; (**c**)—sample S6 with low magnification; and (**d**)—sample S6 with high magnification.

Figure 4 shows the surface morphology of the electrosprayed PTFE with aerogels (sample S6) and without aerogels (sample S0). The deposits were observed to have a structure based on clusters of PTFE and aerogel particle agglomerates formed by the attachment at the deposit of particle agglomerates coming from different electrospray droplets. According to high-magnification SEMs, the electrosprayed particles were mostly in a spherical shape with a rough surface. These particles with different particle sizes, mainly in microscale, gathered and partly overlapped with each other, forming a microporous layer with a relatively compact and disordered stacking structure. The PTFE and aerogel particles travelled with the liquid due to the electrostatic repulsive force. As a result of the low viscosity of the spinning fluid, the emitted jet disintegrated downstream and subsequently broke into charged droplets, PTFE and aerogel particles were encapsulated inside the droplets after the jet breakup. Those droplets underwent solvent evaporation and disrupted to generate smaller droplets containing fewer or even one single particle in one drop since the electrostatic force was sufficiently strong to overcome surface tension [29,30], resulting in a deposited layer composed of PTFE and aerogel particles.

### 3.2. Particle Size Distribution of the Electrosprayed Particles

**Figure 5:**
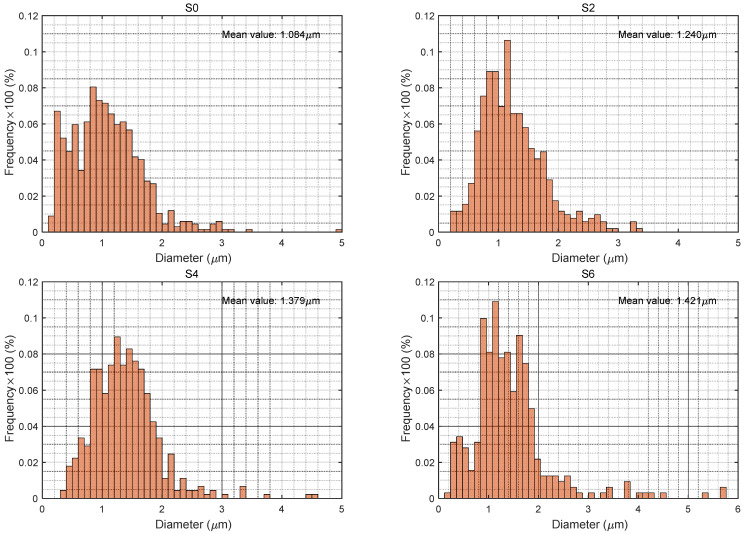
Particle size distribution of the electrosprayed particles.

As seen in Figure 5, the particle sizes of each sample were narrowly distributed ranging from 0.1 to 5.8 μm. Sample S0 with pure PTFE particles, had the lowest average size 1.084 μm. Apparently, applying aerogels in the spinning liquid increased the particles by over 2 μm. The introduction of aerogels changed the nature of the spinning liquid, such as electrical conductivity and surface tension, affecting the formation of the electro-sprayed layer as well as the composition of different particles in the layer. Aerogels slightly increased the electrical conductivity of the spinning liquid, since the surface groups present on aerogels could offer some electrical charges. Meanwhile, the surface tension of the liquid system was reduced because of the balancing force between the liquid and aerogels. Thus, the flow rate was probably increased, the liquid could carry more particles to be deposited on the nonwoven substrate. As the aerogel content applied in spinning liquid increased, the mean particle size of the electrosprayed particles slightly increased since the flow rate and the proportion of larger particles present in the composites increased. However, the value did not increase that much because the PTFE particles, which had smaller dimensions, dominated in the electrosprayed layers.

### 3.3. Structural Characteristics of the Materials

**Figure 6:**
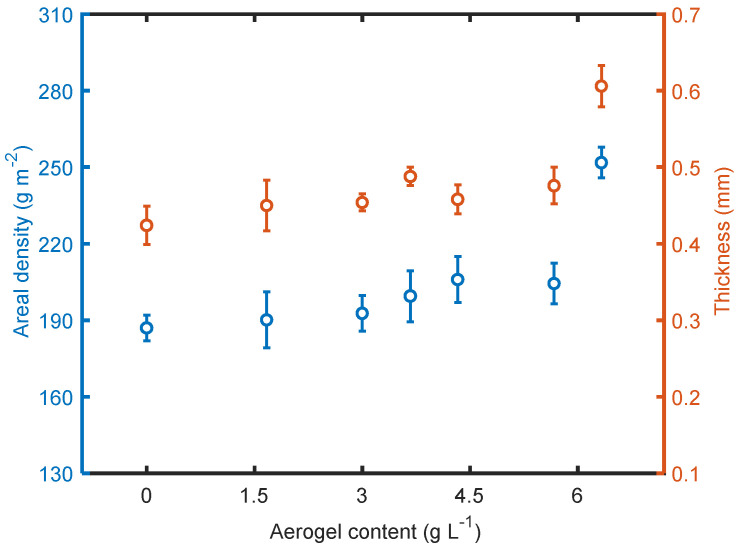
Areal density and thickness of the electrosprayed materials.

The weight of the multilayered materials tended to increase as the aerogel content increased, as seen in Figure 6. This was probably due to the change in liquid nature, which affected the electrospraying process and formation of the electrosprayed microporous materials, as explained above. With the addition of fully hydrophobic aerogels, the conductivity and surface tension of the liquid were altered correspondingly, leading to denser materials. As expected, the thickness of the electrosprayed materials increased with the increase in aerogel content as well. Since the aerogels generally had a larger particle size than a single PTFE particle, aerogels present in the overall structure contributed to thicker materials.

Porosity is an important parameter for a porous or fibrous material because it strongly influences some properties of the material such as filtration, the transmission of air and heat, acoustical properties, etc. As a measure of the void spaces in a material, porosity is a fraction of the volume of voids over the total volume. The total volume porosity P_0_ [–] of layers assembly is defined as

(4) $$P_{0}=1-\frac{w_{T}}{h\cdot \rho_{f}}$$

where *w_T_* [kg m^−2^] *i*s the planar mass (usually in *gsm*/1000, *gsm* is grams of layer per surface area in square meter), *h* [m] is layer thickness, and *ρ_f_* [kg m^−3^] is layer solid phase density. This density is calculated from the expression

(5) $$\rho_{f}={\left(\frac{w_{A}}{\rho_{A}}+\frac{w_{PTFE}}{\rho_{PTFE}}+\frac{w_{PP}}{\rho_{PP}}\right)}^{-1}$$

where *w_A_*, *w_PTFE_*, and *w_PP_* are mass fractions of aerogel, PTFE, and PP, and *ρ_A_* = 120 kg m^−3^, *ρ_PTFE_* = 2200 kg m^−^^3^, _and_ *ρ_PP_* = 920 kg m^−3^ are the corresponding densities.

**Figure 7:**
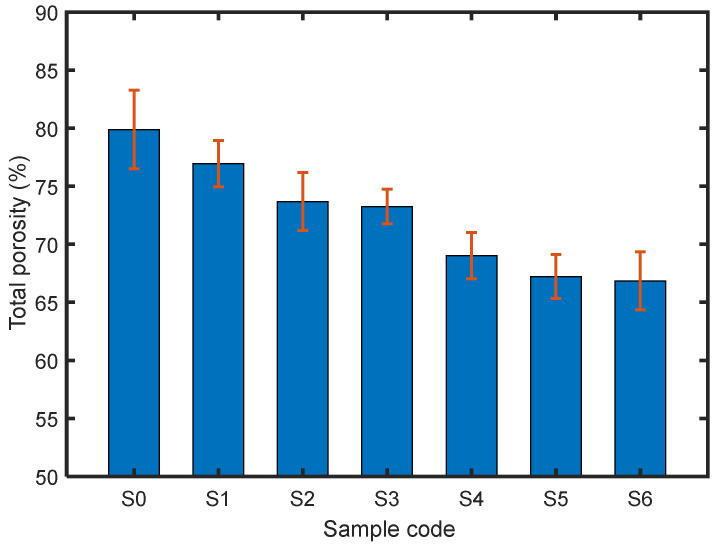
Calculated overall porosity of the materials.

The calculated values of *P*_o_ are shown in Figure 7. The values range from 79% to 68%, varying with the aerogel content. As the aerogel content increased, the calculated total porosity decreased. This is because the deposit morphology is determined by the dynamics of particle arrival to the deposit. The motion of particles approaching the collector involves a combination of a ballistic motion with a certain mean particle velocity *v* and a stochastic motion characterized by a diffusion coefficient *D_p,diff_* [31]. These two parameters together with the particle diameter *D* can be combined in a dimensionless parameter, the P é clet number

(6) $$Pe=\frac{vD}{D_{p,\;diff}}$$

Since the mean particle size increases with the increase in aerogel content, the *Pe* increases as well, the porosity contributed by the agglomerates reaching the substrate decreases [31].

Thus, the porosity of the electrosprayed PTFE materials could be flexibly adjusted by using aerogel as fillers and controlling aerogel filler content, which may be applicable for some cases requiring desirable porosity for a specific application.

### 3.4. Analysis of Surface Roughness

**Figure 8:**
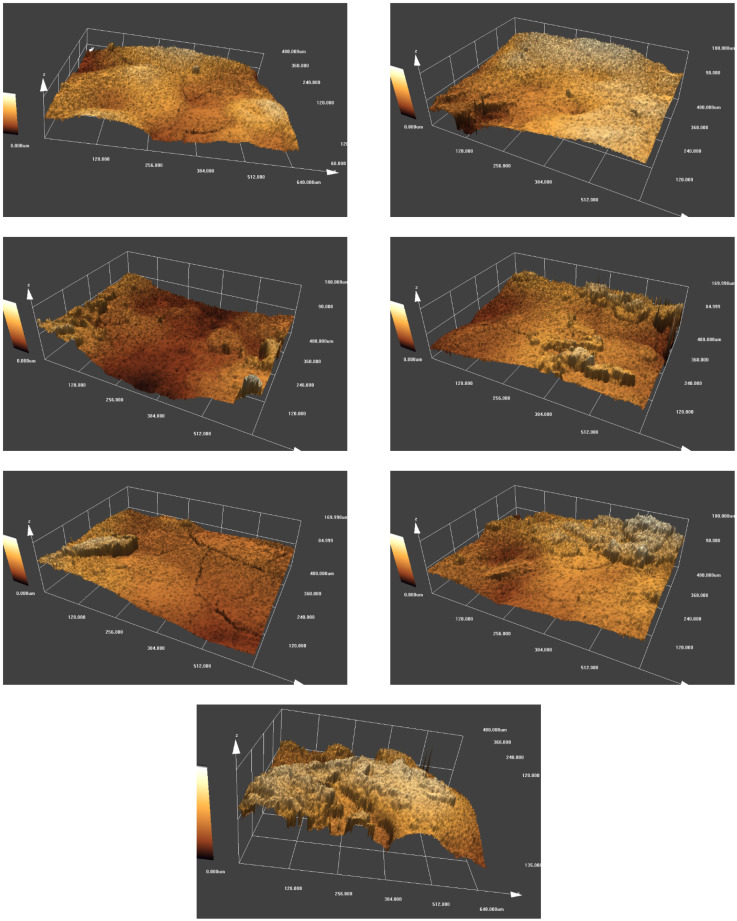
3D surface morphology of the electrosprayed materials.

Figure 8 shows the 3D surface morphology of the electrosprayed materials. On a micrometer scale all the materials had a rough surface with plenty of peaks and valleys. The number of peaks and valleys, the height of peaks, and the depth of valleys differ for each material, depending on the content of aerogel applied. The overall surfaces were flatter for the sample without aerogels (sample S0) or with fewer aerogels (sample S1), while more abrupt peaks and deep valleys appeared on the surface of samples containing more aerogels. As the aerogel content increased, the sharpness of peaks tended to increase as well. The main reason could be that the aerogel particles had a larger dimension in comparison with PTFE particles, the introduction of aerogels brought higher peaks and deeper valleys, thus creating rougher surfaces. With the increase in aerogel content, more peaks and valleys appeared, and the surface roughness of the overall materials increased correspondingly.

The measured arithmetic average height *R_a_*, maximum peak height *R_p_*, maximum valley depth *R_v_*, and skewness *R_sk_* of each material were illustrated in Table 3, allowing a quantitative comparison of surface roughness. *R_a_* increased from 2.7413 μm to 5.2663 μm as the aerogel applied in the spinning liquid increased from 0 to 6.33 g L^−1^, showing significantly improved surface roughness. Considering that a single PTFE particle present in the spinning liquid was around 0.220 μm, and most of the electrosprayed particles were smaller than 2 μm as seen in Figure 5, it was possible to create a smoother surface by controlling the solid concentration of the spinning liquid and spinning parameters. *R_p_*, *R_v_*, and the peak to valley height of the profile were comparable to the aerogel powder size, suggesting that aerogel powder could be filled between these peaks or valleys by modifying the spinning parameters to achieve better performance. Among these materials, sample S6 had a positive value of *R_sk_*, indicating the most peaks on its surface. The positive effect of microscale peaks or valleys lies in the ability to entrap more air inside, providing enhanced hydrophobicity or thermal insulation.

### 3.5. Effect of Aerogels on Surface Hydrophobicity

**Figure 9:**
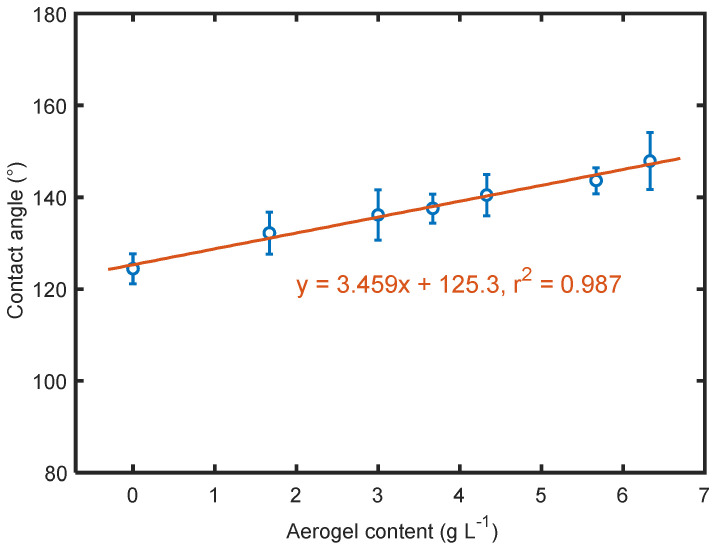
Effect of aerogel content on contact angle.

According to the experimental [32] and simulation results [33], the water contact angle for a smooth PTFE surface was around 108–114°. In this work, the water droplet on the electrosprayed PTFE surface (sample S0) showed a contact angle of approximately 124.44°, 10° more than that of a smooth PTFE surface. This increase was attributed to the surface roughness created by nano spray, allowing more air pockets to be left between the droplet and the material surface. Contact angles of the electrosprayed materials are compared in Figure 9. The contact angles were all over 90°, indicating that all the materials were hydrophobic. Since silica aerogels generally had stronger hydrophobicity than PTFE [34], the contact angle of the electrosprayed PTFE embedded with aerogels increased with the gradual increment in aerogel content. The maximum increase in contact angle was up to 23.44° in this work. A very strong linear relationship between contact angle and aerogel content was observed. This could be mainly due to the introduction of the strong hydrophobic silica aerogel and the gradual increase in the number of hydrophobic groups present on the aerogel surface, which agrees well with previous studies [35,36,37]. In addition, the hydrophobicity of the electrosprayed materials strongly relied on surface roughness, which varied when different contents of aerogels were applied as mentioned above.

### 3.6. Effect of Aerogels on Thermal Conductivity

**Figure 10:**
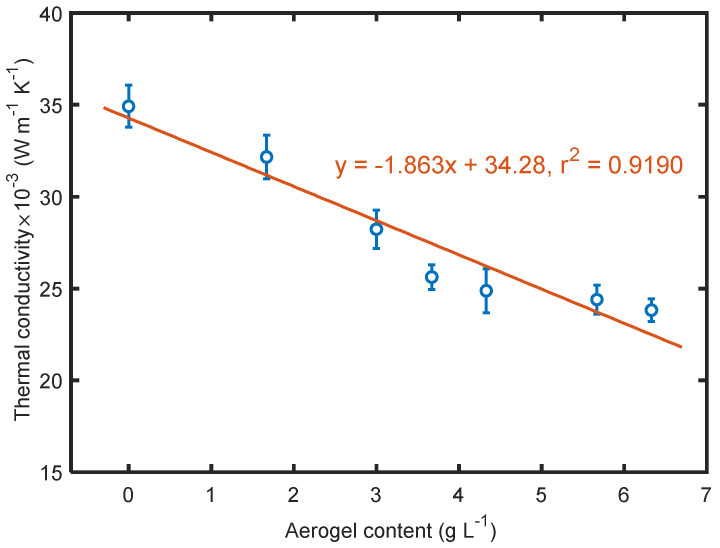
Dependence of thermal conductivity on aerogel content.

As expected, aerogels present in the electrosprayed materials contributed to lower thermal conductivity as seen in Figure 10. The thermal conductivity of the overall structure depended on heat transfer through each component, the PTFE solid, aerogels, and void space in the materials, which entrapped stagnant air. The total thermal conductivity is given by $k_{total}=\sum k_{i}\cdot f_{i}$, where *k_i_* is the thermal conductivity of *i* th component in the material contributing to heat transfer, and *f_i_* is the corresponding volume fraction of that component. Since aerogels have much lower thermal conductivity than PTFE, as the aerogel content increases the proportion of aerogel thermal conductivity *k_aerogel_* increases, causing lower overall thermal conductivity of the material. Especially when the aerogel content was above 4 g/L, the electrosprayed materials achieved very low thermal conductivity close to 0.024 W/(m·K), proving these materials possess superior ability to prevent heat transfer. The relationship between the two parameters shows that thermal conductivity is directly proportional to aerogel content. However, considering the calculated total porosity of each material, it should be noticed that the thermal conductivity of this material was not negatively correlated with total porosity; instead, it tended to decrease with the decrease in total porosity. Generally, the pores in a fibrous material are microscale; in this case, the porosity of the material could be the main factor determining heat transfer rate. However, if the pores are small enough to become comparable to the mean free path of air [38], the cavity could effectively restrict the movement of air particles, causing a significant decrease in thermal conductivity in addition to eliminating convection. Benefitting from a large amount of interconnected nanoscale pores in silica structure, the restriction on heat transfer, which strongly relies on pore size, increased as the aerogel content increased, leading to lower thermal conductivity, while the materials had relatively lower porosity.

## 4. Conclusions

Polytetrafluoroethylene microporous materials composited with aerogels were prepared via the needleless electrospray technique. The obtained materials were characterized in terms of surface morphology, particle size distribution, surface roughness, hydrophobicity, and thermal conductivity of the overall material. It was found that the electrosprayed layers had a relatively compact and disordered stacking structure, consisting of spherical particles with a rough surface. As the aerogel content applied in the spinning liquid increased, the mean particle size of the electrosprayed particles, areal density, and thickness of the multilayered materials increased as well. Surface roughness tended to increase with the increase in aerogel content, surface hydrophobicity was improved by introducing aerogels with a water contact angle up to 147.88°. In addition, the electrosprayed PTFE/aerogel material achieved very low thermal conductivity close to 0.024 W m^−1^ K^−1^), demonstrating a superior ability to prevent heat transfer. By optimizing the spinning liquid and spinning parameters, materials with desirable properties were flexibly prepared, providing a fairly expanded diversity of PTFE/aerogel materials for different applications. It is worth mentioning that the enhanced surface roughness gives access to apply other functional nano or microscale particles to improve a specific property, which might be of interest for further study in this area.

## Figures and Tables

**Table 1:** The electrosprayed materials and details of the spinning solution.

Sample Code of the Electrosprayed Materials	Composition of the Spinning Solution	
	Basic Solution	Aerogel (g L^−1^)
S0	Teflon^TM^ PTFE 30 aqueous dispersion as received	0
S1		1.67
S2		3.00
S3		3.67
S4		4.33
S5		5.67
S6		6.33

**Table 2:** Electrospray parameters for production.

Properties	Value Range
Distance between electrodes (mm)	125
Substrate speed (mm/min)	15
Electrode length (cm)	50
Electrode rotation (rpm)	8
Substrate	Polypropylene spun-bond nonwoven
Voltage (kV)	−10/45
Air flow (m^3^ h)	90/100
Humidity/temperature	40% RH/22 °C

**Table 3:** Some roughness parameters of the electrosprayed materials.

Sample Code	*R_a_* (μm)	*R_p_* (μm)	*R_v_* (μm)	*R_sk_* (μm)
S0	2.7413 ± 0.3916	7.3493 ± 1.6335	13.1058 ± 3.1226	−0.7058 ± 0.6786
S1	2.8363 ± 0.3982	9.0997 ± 3.2011	13.5571 ± 2.0847	−0.6897 ± 0.6868
S2	3.5572 ± 1.1198	11.6905 ± 5.5050	17.9229 ± 6.8362	−0.9110 ± 0.6688
S3	3.6401 ± 0.8821	11.4426 ± 3.2350	14.0517 ± 4.7513	0.0319 ± 0.6267
S4	3.5783 ± 0.5752	12.4228 ± 2.3710	16.6314 ± 4.6911	−0.3828 ± 0.6133
S5	3.7257 ± 1.5401	13.5657 ± 7.7107	18.9747 ± 8.5023	−0.7092 ± 1.0158
S6	5.2663 ± 1.8996	14.4447 ± 9.6368	21.711 ± 7.8459	0.4632 ± 0.6438

## Data Availability

The data presented in this study are available on request from the corresponding author.
